# Antibacterial Activity and Reversal of Multidrug Resistance of Tumor Cells by Essential Oils from Fresh Leaves, Flowers, and Stems of *Montanoa quadrangularis* Schultz Bipontinus (Asteraceae) Collected in Mérida—Venezuela

**DOI:** 10.3390/biom11040605

**Published:** 2021-04-19

**Authors:** Janne Rojas, Gautier Mark-Arthur Ndong Ntoutoume, Patrick Martin, Marielba Morillo

**Affiliations:** 1 Organic Biomolecular Research Group, Faculty of Pharmacy and Bioanalysis, Research Institute, University of Los Andes, Mérida 5101, Venezuela; janner@ula.ve (J.R.); marimorillo@gmail.com (M.M.); 2 ULR7519, Unité Transformations & Agroressources, Université d’Artois-UniLasalle, F-62408 Béthune, France; gautier.ndong@univ-artois.fr

**Keywords:** *Montanoa quadrangularis* Asteraceae, essential oil composition, antibacterial activity, antitumor activity

## Abstract

Essential oils obtained by hydrodistillation of *Montanoa quadrangularis* leaves, flowers, and stems were analyzed by GC and GC/MS techniques revealing myrcene, limonene, β-phellandrene, and sabinene among the main components. The aim of the present study was to evaluate the MDR modulator activity on human MDR1 gene transfected mouse lymphoma cell line and the antimicrobial activity on the essential oils obtained from different parts of the species under investigation. The results revealed that MQL caused a similar increase in the fluorescence activity of the cells at 0.02 μL/mL comparing to the Verapamil^®^ value. The antimicrobial assay was carried out according to the disc diffusion method. Five different bacterial strains (*Staphylococcus epidermidis*, *Bacillus subtilis*, *Pseudomonas aeruginosa*, *Escherichia coli* AG 100, and *Escherichia coli* AG100A) were treated with the essential oils and the zones of inhibition were determined on TSA plates and TSA agar plates supplemented with Tween 20. MQF and MQL showed activity against *B. subtilis*, *S. epidermidis*, and *E. coli* AG 100A while MQS was only active against *E. coli* AG 100A on TSA agar plates experiment. In case of TSA agar plates supplemented with 0.1 *v*/*v*% Tween 20 detergent, MQF showed inhibition on *B. subtilis*, *S. epidermidis*, and *E. coli* AG 100A; MQL was active against *B. subtilis*, *E. coli* AG 100, *and E. coli* AG 100A while MQS was only active against *E. coli* AG 100A.

## 1. Introduction

The Asteraceae family comprises around 32,913 species distributed in 1911 genera and 13 subfamilies; thus, it is considered one of the largest plant families around the world. Many species from the Asteraceae family have been used for medicinal purposes, since these have a wide range of biologically active compounds [1].

*Montanoa* Cerv. in Llave & Lex genus belongs to Heliantheae tribe, Asteraceae Family, and is composed by 75 species found mainly in Mexico and Central America with five species endemics to northern South America [2,3,4]. Species of this family are characterized by producing mainly sesquiterpenes, sesquiterpene lactones, acetylenic compounds, inulin-type fructans, triterpenes, and flavonoids [1,5].

*Montanoa quadrangularis* is distributed through the tropical Andes from Colombia to Venezuela growing at altitudes between 1300 to 2800 m.a.s.l. [6,7,8]. In Venezuela, there are three well identified species; *M. angulata* Badillo, *M. fragans Badillo*, and *M. quadrangularis* Sch. Bip. ex C. Koch [7]. This species is popular and known as “arboloco” or “anime” and is mainly used to recover damaged soil due to deforestation and erosion. Its wood is also used to manufacture handcrafts [7,8].

According to references consulted, *Montanoa* plants have been used in traditional medicine for the biocidal and spermicidal activities, to treat reproductive impairments, for its pro-ejaculatory effects and contraceptive activity [9,10], as well as for its aphrodisiac effect [11]. In this regard, *Montanoa tomentosa* is used in traditional herbal medicine to ease childbirth labor and to cure certain female disorders. Crude extracts of *M. tomentosa* have been reported to have aphrodisiacal effect on male rats. This investigation has pointed out the diterpenes kaurenoic acid, grandiflorenic acid, and monoginoic acid as bioactive molecules showing uterotonic effects [12,13]. Furthermore, the antianxiety effect of *M. tomentosa* was assessed in rats with different hormonal conditions, and low and high hormone levels. The results showed that *M. tomentosa* induced dose-dependent anxiolytic-like actions in rats with low hormone level conditions [14].

Previous phytochemical investigations have reported sesquiterpene lactones such as eudesmanolides and germacrolides as major class of chemical compounds present in *Montanoa* species [9,15,16]. However, flavonoids such as luteolin and apigenin have also been isolated [9]. To the best of our knowledge, there has only been one previous published investigation of *Montanoa quadrangularis* collected from a different location at 1800 m.a.s.l. in Mérida-Venezuela. In this investigation, limonene, sabinene, *trans*-caryophyllene, germacrene-d, and myrcene were identified as major components. Furthermore, the essential oil showed a significant inhibitory effect against Gram-negative bacteria, *Escherichia coli, Klebsiella pneumoniae*, and *Pseudomonas aeruginosa* [17].

The present investigation aims to evaluate the antimicrobial activity and reversal of multidrug resistance of tumor cells by essential oils of leaves, stems, and flowers extracted from *Montanoa quadrangularis* species collected from Mérida-Venezuela.

## 2. Materials and Methods

### 2.1. Plant Material

Fresh aerial parts of *Montanoa quadrangularis* (Asteraceae) were collected in flowering stage in “Los Topes” Farm, Chiguará, Mérida state at 1250 m.a.s.l. A voucher specimen, code AM 16, was deposited in the Dr Luis E. Ruiz T. Herbarium, Faculty of Pharmacy and Bioanalysis, University of Los Andes, Venezuela.

### 2.2. Isolation of Essential Oil

Leaves (1000 g), stems (500 g), and flowers (500 g) were cut into small pieces and subjected to hydrodistillation for 4 h, using a Clevenger-type apparatus. The oils, 2 mL (0.2% *w*/*v* yield, leaves), 0.8 mL (0.16% *w*/*v* yield, stems), and 0.5 mL (0.1% *w*/*v* yield, flowers) were dried over anhydrous sodium sulphate and stored at 4 °C.

### 2.3. Gas Chromatography

GC analysis was carried out with an HP 5890 Series II gas chromatograph (FID, Hewlett-Packard company, Wilmington, DE, USA) using a 30 m × 0.35 mm × 0.25 µm HP-5 fused silica capillary column. The temperature program was from 60 °C to 210 °C at 3 °C.min^−1^, and from 210 °C to 250 °C (2 min hold) at 5 °C.min^−1^. The detector and injector temperature were 250 °C and the carrier gas was N_2_, with split sample introduction.

### 2.4. Gas Chromatography-Mass Spectrometry

This procedure was performed with a Finnigan PolarisQ GC/MS ion trap Bench-top mass spectrometer (Thermo Electron Corporation, Walthman, MA, USA). All conditions were as above, except that the carrier gas was He at a linear velocity of 31.9 cm.s^−1^ and DB-5MS (30 m × 0.25 mm × 0.25 µm) capillary columns were used. The positive ion electron ionization mode was applied, with a mass range of 40–400 amu. Identification of compounds was based on comparisons with published MS data [18] and a computer library search (the database was delivered together with the instrument) and by comparison of their Kovats indices with those of authentic references and with literature values [18]. The identification was confirmed with the aid of authentic samples. Kovats indices were calculated mainly from the GC-MS analysis results [19]. Solvents and other chemicals used were of high purity (analytical grade).

### 2.5. Reversal of Multidrug Resistance of Tumor Cells on L5178 Mouse T-Cell Lymphoma (Transfected with Human MDR1 Gene) by Essential Oils

The L5178 MDR cell line was grown in McCoy’s 5A medium containing 10% heating activated horse serum completed with l-glutamine and antibiotics. The cells were adjusted to a density of 2 × 10^6^ mL, resuspended in serum-free medium, and distributed in 0.5 mL aliquots into Eppendorf centrifuge tubes. The tested oils were added at concentrations of 0.004; 0.04; 0.01; 0.02 μg/mL and incubated for 10 min at room temperature. Verapamil^®^ (10 μg/mL) was used as positive control; 10 µL of Rhodamine 123 (Sigma, St Louis, MO, USA) at final concentration of 5.2 μM was added and cells were incubated for another 20 min at 37 °C. After incubation, cells were washed twice and resuspended in 0.5 mL of phosphate-buffered saline (PBS) to perform the assay. Fluorescence of the cell population was measured by flow cytometry, using a Becton Dickinson FACS can instrument (Becton Dickinson, Franklin Lakes, NJ, USA) [20,21]. The experiments were repeated thrice. MDR1 reversal activity was calculated by the following equation:

$$FAR=\frac{\mathrm{MDR}treated/\mathrm{MDR}control}{P\mathrm{arental}treated/\mathrm{parental}control}$$

### 2.6. Antibacterial Activity of Essential Oils Isolated from Leaves, Stems, and Flowers of Montanoa quadrangularis

The following bacterial strains: *Staphylococcus epidermidis* (ATCC 12228)*, Bacillus subtilis* (ATCC 6633)*, Pseudomonas aeruginosa* (ATCC 9027), *Escherichia* coli AG 100 (ATCC 10536), and *Escherichia* coli AG100A (ATCC 11229), were used in this study.

#### Antimicrobial Method

The antimicrobial assay was carried out according to the disc diffusion method. The strains were maintained in BHI (brain heart infusion) broth at room temperature. Every bacterial inoculum (2.5 mL) was incubated in Mueller-Hinton broth (Britaina^®^ Ref B0213705) at 37 °C for 18 h. The bacterial inoculum was diluted in sterile 0.85% saline to obtain turbidity visually comparable to a McFarland N° 0.5 standard (1 × 10^8^ CFU/mL). Every inoculum was spread over plates containing tryptic soy agar (TSA, Sigma Aldrich) and 0.1 *v*/*v*% Tween 20. The pure essential oils were pipetted on paper discs of 5-mm diameter saturated with 5–10 μL of each sample. The plates were left for 30 min at room temperature and then, incubated at 37 °C for 24 h. The inhibitory zone around the disc was measured and expressed in mm. A positive control was also used to check the sensitivity of the tested organisms using: Ampicillin^®^ (30 µg) and gentamycin^®^ (30 µg); those are reference antibiotics commonly used to treat this kind of bacteria. The minimal inhibitory concentration (MIC) was determined only with microorganisms that displayed inhibitory zones. MIC was determined by dilution of the essential oil in dimethyl sulfoxide (DMSO) and pipetting 10 μL of each dilution onto a filter paper disc. Dilutions of the oil within a concentration range of 5–10 μg/mL were used in the assayed. MIC was defined as the lowest concentration that inhibited the visible bacterial growth [22,23]. A negative control was also included using a filter paper disc saturated with DMSO to check possible activity of this solvent against the bacteria assayed. The experiments were repeated three times.

### 2.7. Statistical Analysis

Experiments were evaluated using three independent assays. Data are expressed as the mean ± standard deviation (SD). Statistical analyses were performed with SPSS (SPSS Inc., Chicago, IL, USA). To determine the statistical significance of differences in activity tested as well as main effects, a one-way analysis of variance (ANOVA) was also performed.

## 3. Results and Discussions

Essential oils from fresh leaves (MQL), stems (MQS), and flowers (MQF) of *Montanoa quadrangularis* were analyzed by GC and GC/MS techniques. A list of identified components along with their percentages of the total oil is given in Table 1. The three samples analyzed showed myrcene (13.5% MQF, 26.2% MQL, 13.6% MQS) and a mixture of limonene + β-phellandrene (27.8% MQF, 16.1% MQL, 12.2% MQS) among the main components; however, trans-sabinol (23.5% MQF), β-caryophyllene (14.7% MQL), and sabinene (22.3% MQS) were also observed in major proportions.

Compounds such as β-selinene (0.7%), viridiflorene (0.3%), *Z*-α-bisabolene (0.6%), and guaiol (1.8%) were only observed in MQF, *E*-3-hexanol (0.7%) was only detected in MQL, while 2-methoxy-*p*-cresol (0.7%) and spathulenol (0.7%) were only present in MQS sample.

Previous investigations carried out with the essential oil of *M. quadrangularis* collected from a different location in Mérida at 1800 m.a.s.l showed limonene (18.83%), sabinene (11.63%), α-pinene (9.90%), and α-tujene (9.27%) as main components from the essential oil of the flowers while the essential oil of the leaves was composed mainly by limonene (18.01%), *trans*-caryophyllene (16.07%), germacrene-d (7.63%), and mircene (6.37%). [15] Additionally, the essential oil of the inforescence of *Montanoa grandiflora* revealed β-phellandrene (6.63%) and α-pinene (2.70%) as major compounds. The essential oil of the stems showed β-phellandrene (7.74%), limonene (4.80%), α-pinene (4.43%), and citronellal (3.50%), while the leaves showed α-pinene (4.27%), β-phellandrene (4.22%), limonene (2.37%), and citronellal (2.82%) as main components [24].

It is well documented that essential oil composition may vary considerably depending on the time of plant collection and are also influenced by environmental conditions at the time of harvesting. In addition, the location where the plant is growing, either sun or shade, may also affect the composition of the oil [25]. Moreover, several reports regarding the variation in the chemical profile of essential oils from a variety of plants collected during different seasons have also been published [26,27,28,29,30].

### 3.1. Reversal of Multidrug Resistance of Tumor Cells on L5178 Mouse T-Cell Lymphoma (Transfected with Human MDR1 Gene) by Essential Oils

The essential oils obtained from different parts (leaves, flowers and stems) of *Montanoa quadrangularis* were investigated for their MDR modulator activity on human MDR1 gene transfected mouse lymphoma cell line. This cell line carries the 170 kDa *P*-glycoprotein in its membrane, which ABC transporter can extrude the chemotherapeutic agent from the cells. Efflux pump-related multidrug resistance is a major obstacle of cancer treatment. Multidrug resistance modulators, which act via the inhibition of this mechanism, seem likely to bring solution for the problem. Verapamil^®^ serves a good model for that purpose; however, the concentration applied as an MDR modulator is beyond the clinically achievable concentration. In the present investigation, cells were stained with Rhodamine 123 fluorescent dye and, after treating the cells with the essential oils, the fluorescence activities were measured by flow-cytometry. According to the results observed (Table 2), only MQL caused a similar increase in the fluorescence activity of the cells at 0.02 μL/mL concentration comparing to the Verapamil^®^ value; however, it is considered a mild activity.

Recent studies suggest that the membrane structure may play a critical role in the MDR activities; the resistance modulator-lipid interaction may be an important factor in the mechanism of drug resistance reversal [20,21]. Since essential oils are made up of highly lipophilic components, the mechanism of action may be attributed to interactions with physiological membrane activities.

### 3.2. Antibacterial Activity of Essential Oils Isolated from Leaves, Stems, and Flowers of Montanoa quadrangularis

The antiseptic properties of aromatic plants and their extracts have been recognized since antiquity and are still used in the medicine, food, and cosmetic industry. In the present experiment, *M. quadrangularis* essential oils were investigated for their antibacterial properties in the agar disc diffusion assay. Five different bacterial strains were treated with the essential oils and the zones of inhibition were determined on TSA plates and TSA agar plates supplemented with Tween 20. MQF and MQL showed activity against *B. subtilis*, *S. epidermidis*, and *E. coli* AG 100A, while MQS was only active against *E. coli* AG 100A on TSA agar plates experiments (Table 3).

In the case of TSA agar plates supplemented with 0.1 *v*/*v*% Tween 20 detergent, MQF showed inhibition on *B. subtilis*, *S. epidermidis*, and *E. coli* AG 100A. MQL was active against *B. subtilis*, *E. coli* AG 100, *and E. coli* AG 100A while MQS was only active against *E. coli* AG 100A. However, *P. aeruginosa* and *E. coli* AG 100 were resistant to all tested essential oils (Table 4).

The proton pump deleted mutant, *E. coli* AG 100A was susceptible to the tested essential oils, which supports that certain components of the oil complexes may be substrates of the bacterial membrane transport systems. Tween 20 was given to the agar plates to increase the solubility of essential oils.

Previous investigations have reported the antimicrobial activity of Asteraceae species against bacterial pathogens. Compounds like humulene, isohumullene, caryophyllene, and germacrene-d, among others, have shown bacterial and fungal growth inhibition. Since preventing recurrent bacterial infections and improving patients’ quality of life have been the goals of many research groups, natural products with potential antimicrobial activity might be considered as a viable alternative for the effective treatment of diseases that are caused by these microorganisms [31,32,33].

The mechanism of action of essential oil components is not fully understood, it is assumed that membrane disruption by the lipophilic components is involved in the antibacterial action. Certain components of essential oils can act as uncouplers, which interfere with proton translocation over a membrane vesicle and subsequently interrupt ADP phosphorylation. Specific terpenoids with functional groups, e.g., phenolic, alcohols or aldehydes, also interfere with membrane-integrated or associated enzyme-proteins, stopping their production or activity [22]. The mode of action also depends on the microorganism and is mainly related to its cell wall structure. Gram-negative bacteria have intrinsic resistance against toxic components, since they have a permeability barrier against toxic agents. Hydrophobic macromolecules, such as essential oil constituents, are unable to penetrate the barrier. On the other hand, essential oils usually express low aqueous solubility, which prevents them from reaching a toxic level in cellular membranes [22,30].

## 4. Conclusions

Essential oils of flowers, leaves, and stems analyzed in the present investigation showed myrcene, limonene, β-phellandrene, trans-sabinol, β-caryophyllene, and sabinene as main components. Regarding the reversal of multidrug resistance of tumor cells assay, MQL caused an increase in the fluorescence activity of the cells at 0.02 μL/mL. According to references consulted, the membrane structure may play a critical role in the MDR activities, the resistance modulator–lipid interaction may be an important factor in the mechanism of drug resistance reversal. In addition, MQF and MQL showed activity against *B. subtilis*, *S. epidermidis*, and *E. coli* AG 100A, while MQS was only active against *E. coli* AG 100A.

## Figures and Tables

**Table 1:** Chemical composition of the essential oil of flowers, leaves, and stems of *Montanoa quadrangularis* collected in Mérida- Venezuela.

Compounds *	RI	MQF (%)	MQL (%)	MQS (%)
*E*-3-hexanol	852	-	0.7	-
santolina triene	908	1.2	0.3	-
α-thujene	927	3.1	5.2	9.2
α-pinene	934	1.9	1.9	4.5
Sabinene	974	2.6	8.9	22.3
β-pinene	978	0.8	3.5	9.9
Myrcene	991	13.5	26.2	13.6
*p*-cymene	1025	0.4	0.2	0.8
limonene + β-phellandrene	1029	27.8	16.1	12.2
1,8-cineol	1032	2.7	5.7	-
γ-terpinene	1058	-	0.3	1.3
trans-sabinol	1140	23.5	4.8	0.8
terpin-4-ol	1178	1.3	1.1	5.8
2-methoxy-*p*-cresol	1187	-	-	0.7
α-terpineol	1192	0.4	0.6	-
β-elemene	1393	1.2	0.3	-
β-caryophyllene	1420	5.9	14.7	1.3
α-humulene	1455	0.4	0.7	-
germacrene D	1482	1.8	3.2	5.7
β-selinene	1487	0.7	-	-
viridiflorene	1496	0.3	-	-
*Z*-α-bisabolene	1506	0.6	-	-
δ-cadinene	1525	0.5	0.2	-
germacrene B	1555	0.5	1.3	-
spathulenol	1578	-	-	0.7
caryophyllene oxide	1584	1.6	1.2	3.3
khusimone	1591	0.4	1.2	0.5
Guaiol	1597	1.8	-	-

* The composition of the essential oil was determined by comparison of the mass spectrum of each component with Wiley GC/MS library data and from its retention index (RI), Retention time (RT).

**Table 2:** Fluorescence activity ratios (FAR) tested for *Montanoa quadrangularis* essential oils.

Samples	Experiment I.		Experiment II.	
Verapamil (10 μg/mL)	3.18 ± 0.02		12.81 ± 0.04	
μL/mL	0.004	0.04	0.01	0.02
MQF	1.06 ± 0.02 ^b^	1.94 ± 0.0 ^d^	-	1.24 ± 0.01 ^c^
MQL	0.91 ± 0.02 ^b^	1.67 ± 0.0 ^c^	-	8.26 ± 0.01 ^d^
MQS	-	3.27 ± 0.0 ^d^	1.93 ± 0.02 ^b^	2.02 ± 0.01 ^c^
DMSO K (40 μL/mL)	-	0.92 ± 0.01	-	0.89 ± 0.01

MQF: *M. quadrangularis* essential oil of flowers, MQL: *M. quadrangularis* essential oil of leaves, MQS: *M. quadrangularis* essential oil of stems. *p* < 0.05 ^b,c,d^ different letters represent the statistical difference between dates.

**Table 3:** Zones of inhibition (mm) of essential oils on TSA agar plates.

Sample V(µL)	*S. epidermidis*		*B. subtilis*		*P. aeruginosa*		*E. coli* AG 100		*E. coli* AG100A	
	5	10	5	10	5	10	5	10	5	10
MQF	8 ± 0.1 ^c^	9 ± 0.06 ^d^	10 ± 0.1 ^e^	10 ± 0.2 ^e^	0 ± 0.0	0 ± 0.0	0 ± 0.0	0 ± 0.0	7 ± 0.0 ^b^	10 ± 0.06 ^e^
MQL	0 ± 0.0 ^a^	8 ± 0.1 ^c^	9 ± 0.1 ^d^	10 ± 0.06 ^e^	0 ± 0.0	0 ± 0.0	0 ± 0.0	0 ± 0.0	0 ± 0.0	7 ± 0.0 ^b^
MQS	0 ± 0.0 ^a^	0 ± 0.0 ^a^	0 ± 0.0 ^a^	0 ± 0.0 ^a^	0 ± 0.0	0 ± 0.0	0 ± 0.0	0 ± 0.0	0 ± 0.0 ^a^	10 ± 0.02 ^e^
Ampicillin^®^	10 ± 0.06		9 ± 0.1		20 ± 0.1		11 ± 0.2		18 ± 0.1	
Gentamicin^®^	28 ± 0.1		22 ± 0.0		21 ± 0.1		24 ± 0.0		21 ± 0.1	

*p* < 0.05 ^a,b,c,d,e^. Different letters represent the statistical difference between dates.

**Table 4:** Zones of inhibition (mm) on TSA agar plates supplemented with 0.1v/v % Tween 20 detergent.

Sample V (μL)	*S. epidermidis*		*B. subtilis*		*P. aeruginosa*		*E. coli* AG 100		*E. coli* AG100A	
	5	10	5	10	5	10	5	10	5	10
MQF	8 ± 0.06 ^b^	12 ± 0.1 ^c^	0 ± 0.0 ^a^	8 ± 0.06 ^b^	0 ± 0.0	0 ± 0.0	0 ± 0.0 ^a^	0 ± 0.0	9 ± 0.2 ^b^	12 ± 0.06 ^c^
MQL	0 ± 0.0	0 ± 0.0	8 ± 0.06 ^b^	8 ± 0.2 ^b^	0 ± 0.0	0 ± 0.0	0 ± 0.0 ^a^	7 ± 0.1 ^b^	0 ± 0.0 ^a^	8 ± 0.05 ^b^
MQS	0 ± 0.0	0 ± 0.0	0 ± 0.0	0 ± 0.0	0 ± 0.0	0 ± 0.0	0 ± 0.0	0 ± 0.0	0 ± 0.0 ^a^	12 ± 0.06 ^c^
Ampicillin^®^	16 ± 0.06		13 ± 0.1		20 ± 0.06		13 ± 0.0		18 ± 0.1	
Gentamicin^®^	35 ± 0.1		28 ± 0.1		23 ± 0.1		20 ± 0.06		21 ± 0.1	

*p* < 0.05 ^a,b,c^. Different letters represent the statistical difference between dates.

## Data Availability

All the results used in this work to support the conclusions of this study are included in the article.

## References

[1] Rustaiyan A., Faridchehr A. (2021). Constituents and biological activities of selected genera of the Iranian Asteraceae family. J. Herb. Med..

[2] Funk V.A. (1982). The systematics of *Montanoa* (Asteraceae, Heliantheae). Mem. N. Y. Bot. Garden..

[3] Plovanicha A.E., Panerob J.L. (2004). A phylogeny of the ITS and ETS for *Montanoa* (Asteraceae: Heliantheae). Mol. Phylogenet. Evol..

[4] Robles-Zepeda R.E., Molina-Torres J., Lozoya-Gloria E., López M.G. (2005). Volatile organic compounds of leaves and flowers of *Montanoa tomentosa*. Flavour Fragr. J..

[5] Bessada S.M.F., Barreira J.C.M., Oliveira M.B.P.P. (2015). Asteraceae species with most prominent bioactivity and their potential applications: A review. Ind. Crop. Prod..

[6] Badillo V. (2001). Suplemento a la lista de las Asteraceae de Venezuela. Ernstia.

[7] Fersain A. (1999). Maravillas vegetales: El Arboloco o pauche. Centro Int. Orgánica.

[8] Álvarez L. (2003). Biología, uso y Manejo del Arboloco (Montanoa quadrangularis).

[9] El-Toumy S., Omara E., Nada S., Bermejo J. (2011). Flavone C-glycosides from *Montanoa bipinnatifida* stems and evaluation of hepatoprotective activity of extract. J. Med. Plants.

[10] Carro-Juárez M., Lobatón I., Benítez O., Espíritu A. (2006). Pro-ejaculatory effect of the aqueous crude extract of cihuapatli (*Montanoa tomentosa*) in spinal male rats. J. Ethnopharmacol..

[11] Sumalatha K., Saravana K., Mohana L. (2010). Review on natural aphrodisiac potentials to treat sexual dysfunction. Int. J. Pharm. Ther..

[12] Villa-Ruano N., Betancourt-Jiménez M.G., Lozoya-Gloria E. (2010). cDNA isolation and gene expression of kaurene oxidase from Montanoa tomentosa (zoapatle). Rev. Latinoam. Química.

[13] Villa-Ruano N., Betancourt-Jiménez M.G., Lozoya-Gloria E. (2009). Biosynthesis of uterotonic diterpenes from *Montanoa tomentosa* (Zoapatle). J. Plant Physiol..

[14] Estrada-Camarena E., Sollozo-Dupont I., Islas-Preciado D., González-Trujano M.E., Carro-Juárez M., López-Rubalcava C. (2019). Anxiolytic- and anxiogenic-like effects of Montanoa tomentosa (Asteraceae): Dependence on the endocrine condition. J. Ethnopharmacol..

[15] Braca A., Cioffi G., Morell I., Venturella F., Pizza C., De Tommasi N. (2001). Two new sesquiterpene lactones from *Montanoa tomentosa* ssp. microcephala. Planta Med..

[16] Müller S., Murillo R., Castro V., Brecht V., Merfort I. (2004). Sesquiterpene lactones from *Montanoa hibiscifolia* that inhibit the transcription factor NF-κB. J. Nat. Prod..

[17] González F., Hernández-Molina F., Rojas-Fermin L., Araque M. (2015). Composición química y evaluación in vitro de la actividad antibacteriana del aceite esencial de *Montanoa quadrangularis* Sch. Bip. ex C. Koch. contra cepas bacterianas de referencia internacional. Ciencia.

[18] Adams R.P. (1995). Identification of Essential Oil Components by GC/MS.

[19] Davies N. (1990). Gas chromatographic retention indices of monoterpenes and sesquiterpenes on methyl silicone and carbowax 20 M. phases. J. Chromatogr. A.

[20] Gyémánt N., Tanaka M., Molnár P., Deli J., Mándoky L., Molnár J. (2006). Reversal of multidrug resistance of cancer cells in vitro: Modification of drug resistance by selected carotenoids. Anticancer Res..

[21] Rauf A., Uddin G., Raza M., Ahmad B., Jehan N., Siddiqui B.S., Molnar J., Csonka A., Szabo D. (2016). Reversal of Multidrug Resistance in Mouse Lymphoma Cells by Extracts and Flavonoids from *Pistacia integerrima*. Asian Pac. J. Cancer Prev..

[22] Putri H.I., Lauda F.Y.M. (2019). Phytochemical screening and in vitro antibacterial activity of sweet basil leaves (*Ocimum basilicum* L.) essential oil against Cutibacterium acnes ATCC 11827. AIP Conf. Proc..

[23] Khalil N., Ashour M., Fikry S., Singab N.A., Salama O. (2017). Chemical composition and antimicrobial activity of the essential oils of selected Apiaceous fruits. Future J. Pharm. Sci..

[24] Pérez-Amador M.C., Muñoz V., Noyola A., García-Jiménez F. (2006). Essential oil and phototoxic compounds in *Clibadium surinamense* L. and *Montanoa grandiflora* D.C. (Asteraceae). Int. J. Exp. Bot..

[25] Juliani H.R., Karoch A.R., Juliani H.R., Trippi V.S., Zygadlo J.A. (2002). Intraspecific variation in the leaf oils of *Lippia junelliana* (Mold.). Biochem. Syst. Ecol..

[26] Hussain A.I., Anwar F., Sherazi S.T.H., Przybylski R. (2008). Chemical composition antioxidant and antimicrobial activities of basil (*Ocimum basilicum*) essential oils depends on seasonal variations. Food Chem..

[27] Celiktas O.Y., Kocabas H.E.E., Bedir E., Sukan V.F., Ozek T., Baser K.H.C. (2007). Antimicrobial activities of methanol extracts and essential oils of *Rosmarinus officinalis*, depending on location and seasonal variations. Food Chem..

[28] Van Vuuren S.F., Viljoen A.M., Ozek T., Demirici B., Baser H.C. (2007). Seasonal and geographical variation of *Heteropyxis natalensis* essential oil and the effect thereof on the antimicrobial activity. S. Afr. J. Bot..

[29] Rojas J., Morales A., Pasquale S., Márquez A., Rondón M., Veres K., Máthé I. (2006). Comparative study of the chemical composition of the essential oil of *Lippia oreganoides* L. collected in two different seasons in Venezuela. Nat. Prod. Commun..

[30] Karpiński T.M. (2020). Essential Oils of Lamiaceae Family Plants as Antifungals. Biomolecules.

[31] Abad M.J., Bedoya L.M., Bermejo P., Rai M.K., Kon K.V. (2013). Essential Oils from the Asteraceae Family Active against Multidrug-Resistant Bacteria. Fighting Multidrug Resistance with Herbal Extracts. Fighting Multidrug Resistance with Herbal Extracts.

[32] Dias H.J., Vieira T.M., Carvalho C.E., Aguiar G.P., Wakabayashi K.A.L., Turatti I.C.C., Willrich G.B., Groppo M., Cunha W.R., Martins C.H.G. (2017). Screening of Selected Plant-Derived Extracts for Their Antimicrobial Activity against Oral Pathogens. Int. J. Complement. Altern. Med..

[33] Chiavari-Frederico M.O., Barbosa L.N., Carvalho dos Santos I., Ratti da Silva G., Fernandes de Castro A., de Campos Bortolucci W., Wietzikoski Lovato E.C. (2020). Antimicrobial activity of Asteraceae species against bacterial pathogens isolated from postmenopausal women. PLoS ONE.

